# Collection Efficiencies of High Flow Rate Personal Respirable Samplers When Measuring Arizona Road Dust and Analysis of Quartz by X-ray Diffraction

**DOI:** 10.1093/annhyg/met075

**Published:** 2014-01-27

**Authors:** Peter Stacey, Taekhee Lee, Andrew Thorpe, Paul Roberts, Gillian Frost, Martin Harper

**Affiliations:** 1.Analytical Sciences Unit, Health and Safety Laboratory, Harpur Hill, Buxton SK17 9JN, UK;; 2.Exposure Assessment Branch, Health Effects Laboratory Division, National Institute for Occupational Safety and Health, Morgantown, WV 26505, USA;; 3.Occupational Hygiene Unit, Health and Safety Laboratory, Harpur Hill, Buxton SK17 9JN, UK;; 4.Mathematical Sciences Unit, Health and Safety Laboratory, Harpur Hill, Buxton SK17 9JN, UK

**Keywords:** Arizona road dust, CIP 10 R, FSP10, GK2.69, quartz, respirable, sampler, silica, SIMPEDS, x-ray diffraction, XRD

## Abstract

Prolonged exposure to respirable crystalline silica (RCS) causes silicosis and is also considered a cause of cancer. To meet emerging needs for precise measurements of RCS, from shorter sampling periods (<4h) and lower air concentrations, collaborative work was done to assess the differences between personal respirable samplers at higher flow rates. The performance of FSP10, GK2.69, and CIP 10 R samplers were compared with that of the Safety In Mines Personal Dust Sampler (SIMPEDS) sampler as a reference, which is commonly used in the UK for the measurement of RCS. In addition, the performance of the FSP10 and GK 2.69 samplers were compared; at the nominal flow rates recommended by the manufacturers of 10 and 4.2 l · min^−1^ and with flow rates proposed by the National Institute for Occupational Safety and Health of 11.2 and 4.4 l · min^−1^. Samplers were exposed to aerosols of ultrafine and medium grades of Arizona road dust (ARD) generated in a calm air chamber. All analyses for RCS in this study were performed at the Health and Safety Laboratory. The difference in flow rates for the GK2.69 is small and does not result in a substantial difference in collection efficiency for the dusts tested, while the performance of the FSP10 at 11.2 l · min^−1^ was more comparable with samples from the SIMPEDS. Conversely, the GK2.69 collected proportionately more crystalline silica in the respirable dust than other samplers, which then produced RCS results most comparable with the SIMPEDS. The CIP 10 R collected less ultrafine ARD than other samplers, as might be expected based on earlier performance evaluations. The higher flow rate for the FSP10 should be an added advantage for task-specific sampling or when measuring air concentrations less than current occupational exposure limits.

## INTRODUCTION

A key tool, used to assess the exposure of workers to hazardous dusts and the effectiveness of controls, is to obtain personal samples of dust over a specific period of work and then to measure for the mass of a hazardous substance. Mineral dusts associated with various pneumoconioses are often measured in terms of the respirable fraction, which is the range of particle size diameters that can penetrate to the alveolar region of the lungs. Prolonged exposure to respirable crystalline silica (RCS) causes silicosis and is also considered a cause of cancer (IARC, 1997). There are several crystalline forms of silica and the most commonly encountered are quartz and cristobalite. Quartz is studied in this paper because it is found in many natural materials and is a hazard in many large industry sectors, including mining/quarrying, construction, brick and tile manufacturer, foundries, and stonemasonry. Exposure to cristobalite occurs less frequently and is found when quartz or amorphous silica is heated, usually in an industrial process. Compliance with exposure limits requires the collection of respirable dust to determine an individual’s exposure to RCS. Respirable particles are a health-related size fraction defined by the International Organisation for Standardisation document 7708 (ISO 7708, 1995) and the European Norm (EN) 481 (CEN, 1993) as particles generally <15 μm diameter with a mass median aerodynamic diameter of 4.0 μm, in respect to all airborne particles. Respirable dust is sampled from the airborne aerosol using a cyclone or impactor to separate the required size fraction from larger particles in the aerosol. It should be demonstrated by manufacturers or others that samplers can meet a size classification by challenging them to aerosols of particles, each with a different median size and then assessing the resultant bias for reasonably likely distributions against a standard convention. No sampler matches the respirable convention exactly and differences in performance can cause differences in the air concentrations recorded by the Occupational Hygienist. Differences between samplers, at particular workplaces, may cause consistent bias rather than random variation, which, if significant, may lead to differences in interpretation and decision making that ultimately impact on costs to industry and society, health of the worker, and the assessment of controls.

As the understanding of the health effect of exposure to RCS has improved, lower occupational exposure limits have been proposed at levels that approach the limit of the capabilities of the instrumental techniques. The difficulties of obtaining accurate measurements for short-term sampling (<4h) or measuring air concentrations of airborne particles <0.05 mg·m^−3^ with air samplers of flow rates <4 l · min^−1^ routinely employed for collecting RCS have been discussed (Stacey, 2007). To meet the emerging needs, newer samplers were investigated that can run at higher flow rates >4 l · min^−1^ (Stacey and Thorpe, 2010; Lee *et al.*, 2010, 2012). If Occupational Hygienists are to use such samplers routinely then it is important to understand the relative differences between emerging and current sampler designs. Previous work (Lee *et al.*, 2010) compared the performance of the FSP10 and GK 2.69 samplers at the manufacturer’s recommended flow rates (10 and 4.2 l · min^−1^) and concluded that higher flow rates (11.2 and 4.4 l · min^−1^) were needed to comply with the ISO respirable fraction definition more closely. Lee *et al.* (2012) compared the mass differences obtained with the GK 2.69 and the FSP10 when exposed to coal dust at these new suggested flow rates with the Dorr Oliver and BGI4L samplers (based on a Higgins–Dewell design) which showed that the mass concentration of respirable dust from the FSP10 sampler was considerably higher.

This paper describes collaborative work between the Health and Safety Laboratory (HSL) and the National Institute for Occupational Safety and Health (NIOSH) to assess the differences in gravimetric and RCS measurements when challenging the newer high flow rate samples currently available to airborne concentrations of two grades of Arizona road dust (ARD) generated in a calm air chamber.

## MATERIALS AND METHODS

The experimental protocol used previously in a study of 13 respirable samplers (Stacey *et al.*, 2013) was again used in this work. These high flow rate samplers were exposed to ultrafine and medium ARD at different recommended flow rates. The results were compared against those obtained by the Safety In Mines Personal Dust Sampler (SIMPEDS), commonly employed in the UK for measurement of RCS. This work also included the evaluation of new Parallel Particulate Impactor (PPI) (SKC Ltd) operating at 8 l · min^−1^; however, it was discovered that two of the units supplied were not correctly machined, so their results were excluded from this work.

### Respirable samplers

The SIMPEDS (Casella Measurement, Bedford, UK) operating at a flow rate of 2.2 l·min^−1^ was used in every test for comparison. This sampler was characterized previously by Maynard and Kenny (1995) and is frequently used in the UK for RCS sampling. The high volume samplers evaluated included: the FSP10 [Gesellschaft für Schadstoffmesstechnik (GSM) GmbH (now GSA Messgerätebau), Neuss, Germany] operating at the manufacturer’s quoted flow rate of 10 l·min^−1^ and at the NIOSH revised flow rate of 11.2 l·min^−1^, the GK 2.69 (BGI Inc., Waltham, MA, USA) operating at the manufacturer’s flow rate of 4.2 l·min^−1^ and the NIOSH-proposed flow rate of 4.4 l·min^−1^, and the CIP10 R (Arelco ARC, Paris, France) operating at its recommended flow rate of 10 l·min^−1^. The SIMPEDS sampler was made of conductive plastic and the GK2.69 and FSP10 samplers were metal.

### Aerosol chamber

Aerosols were generated in the calm air dust chamber (developed at HSL), which has been documented in papers by Thorpe and Walsh (2007) and Stacey and Thorpe (2010) and was used for a recent study of 13 lower flow rate respirable samplers conducted at HSL. The chamber is optimized to produce uniform aerosols, a prerequisite for sampler evaluation studies. The performance of the apparatus is discussed in Stacey *et al.* (2013). The sampler testing system consists of two boxes 1×1 × 1 m, one placed on top of the other. Dust is generated at the top and the samplers are placed in the bottom chamber. The system used at HSL is large enough to accommodate 15 samplers that are rotated during sampling to improve uniformity of results. Air is drawn down through the system past the samplers. Samplers were tested in calm air conditions and wind speeds in the chamber during the tests were ~0.4 cm·s^−1^. Low air flow rates <0.3 m·s^−1^ are typical of most, but not all indoor rates of air movement (Baldwin and Maynard, 1998). Although the temperature and relative humidity inside the chamber were not regulated, they were fairly constant between 21 and 23°C and 30–35%, respectively, throughout the tests. The dust was introduced into the chamber using the rotating brush generator model RBG 1000 manufactured by PALAS GmbH.

### Experimental approach

Three replicates of each high flow rate sampler type were placed on the rotating table in the aerosol chamber with three SIMPEDS reference samplers. The maximum capacity of the system was for 15 samplers (five sets of three samplers of each type). Not all sampler types could be fitted in the chamber at the same time, so combinations of triplicate samplers were sampled. Each run included three SIMPEDS samplers to compare different runs. The air concentration value obtained by each sampler type could then be compared with either the average air concentration of respirable dust obtained by all samplers in each run or the average value obtained by the three SIMPEDS. Three loading levels from each dust with similar air concentrations and three loading levels from each dust with different air concentrations were collected for each sampler type. The air concentration of dust in the chamber was monitored using a Microdust Pro (Casella Ltd) real-time dust monitor. The intention was to achieve loadings on the lowest flow rate SIMPEDS sampler of between 0.18 and 1.20mg, so that the influence of the imprecision of gravimetric analysis on any findings would be negligible. The sampling times were short and lasted from 30 to 120min. The flow rate of the samplers was calibrated using a TSI 4046/4116 primary calibrator (TSI Inc., Shoreview, MN, USA). None of the samplers were tested for potential leaks if they had not been found during the initial calibration stage which, while not an ideal practice, was thought to provide a realistic indication of routine performance. The stability of the flow rate was visually checked during and measured before and after each run, which should have identified any flow rate issue, potentially due to leakage. Collection media were conditioned in a room with controlled humidity (50±5%) and temperature 20±2°C. Filters were weighed using an ultra-microbalance (UX6; Mettler Toledo, Columbus, OH, USA) with a possible resolution of 0.1 μg, although the resolution was set to 1 μg for these experiments.

### Challenge test dusts

Both ‘ultra fine’ and ‘medium’ grades of ARD (ISO 12103-1, 1997), with a particle size between 0–10 and 0–80 μm, were used for these tests as they are standard materials that contain a significant percentage of crystalline silica. When aerosolized, these dusts produced aerosols in the calm air test chamber with mass median aerodynamic diameters of 2.8 and 4.6 μm, respectively, when measured using a Marple cascade impactor. The Marple cascade impactor determines the proportion of the size-fractionated dust collected for each of nine stages gravimetrically. The particle size distributions of the aerosolised ARD powders are shown in Fig. 1.

**1:**
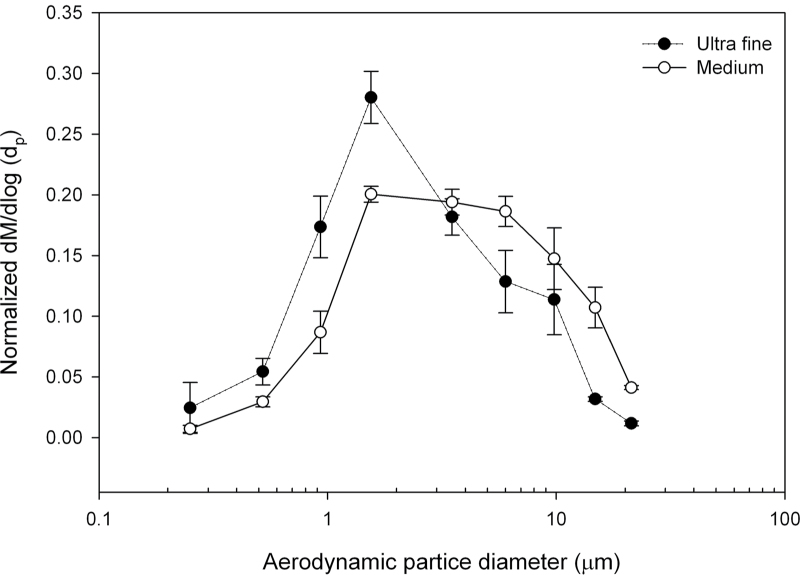
Particle size distribution of aerosolised ARD test dust.

### Analytics

The silver filters (0.8-μm pore size) from the SIMPEDS were analysed using a direct on-filter approach based on MDHS 101 (HSE, 2005). Silver was selected because it is very weight stable. The 37-mm diameter 5-μm pore size polyvinylchloride filters in the GK2.69 samplers were analysed following NIOSH method 7500 (NIOSH, 2003). The 8-μm pore size cellulose nitrate filters from FSP10 samplers were prepared for RCS analysis using an in-house method based on one used at Institut fuer Arbeitsschutz in Germany. This involves wetting the air sample filter in 1,3 butadione (CAS 107-88-0), before placing it in a furnace and heating from room temperature to 450°C to ash the filter. After 4h the crucible was allowed to cool and placed in a beaker. The crucible and residue were then ultrasonicated for 5 mins with isopropanol and the residue filtered onto a silver filter for instrumental analysis. Samples of dust collected on foams in the CIP 10 R were analysed following the Association Française de Normalisation (AFNOR) method NF X 43–295 (AFNOR, 1995). A small modification was made to the method to ensure that, after the removal from the furnace, both the sample and crucible were ultrasonicated in isopropanol before filtration onto the analysis filter. All analyses in this work were performed at HSL.

### Statistical analysis

These data were examined in three ways following a similar protocol discussed in Stacey *et al.* (2013). Firstly, for comparability with Stacey *et al.* (2013), we compared the slopes of the straight line relationships between mean air concentrations (gravimetric results/air volume). For each run, the mean air concentration calculated from all the samplers was compared with the measured air concentration for each of the three samplers of each sampler type. The mean air concentration value should represent a relatively unbiased estimate of the ‘true’ concentration in these experiments, as the same group of samplers were not used in every run and each run contained samplers that performed higher and lower than expected. Two mixed effects models, one for each dust type, were used to compare the air concentration values from each sampler type (mean respirable dust concentration and each sampler value in each run) over the whole range of dust concentrations generated. The sampling run was entered as a random effect, with the mean air concentration and the interaction between the mean air concentration and sampler type entered as a fixed effect. The intercept term (constant term) was constrained to zero, so the estimated slopes would provide an estimate of the relative difference. The 95% confidence intervals for the slopes obtained for each sampler type were then compared against each other to determine the magnitude of their similarity. Standard errors were estimated using non-parametric bootstrapping, and comparisons of the relative differences obtained for each sampler were made using the Wald test, with adjustment for multiple comparisons using Sidak’s procedure. Statistical evaluations were performed using Stata Statistical Software (StataCorp, 2011). Additionally, the air concentration of respirable dust for each sampler when compared with the mean of the triplicate SIMPEDS sampler values in each run were then examined for each dust to demonstrate the similarity of the performance of the SIMPEDS for each sampler type and flow rate.

Secondly, the ratios of the mass of RCS in the respirable dust were then investigated to assess any changes in the performance of the samplers in collecting the particle size distribution of silica in the aerosol.

Thirdly, the overall differences in RCS concentrations, compared to those collected by the SIMPEDS were investigated. Two-sided *t*-tests were used to compare the significance of ratios of RCS with respirable dust and the RCS concentration from the SIMPEDS. We have presented the Figs 3 and 4 and regression values comparing the performance of the SIMPEDS sampler with the respirable dust air concentration values obtained by each sampler for both dusts in Table 3 in a format similar with the work of Lee *et al.* (2012) to allow a comparison. Typically, 12 samples of each high volume sampler design were analysed for RCS at HSL. Each RCS measurement was compared with the average RCS concentration obtained from three replicate SIMPEDS included in each run. In all, there were 108 measurements for the SIMPEDS sampler included in the comparison over all runs.

**3:**
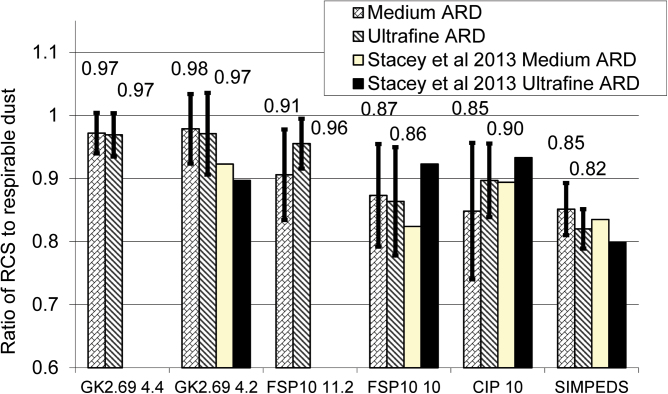
Ratio of RCS to respirable dust.

**4:**
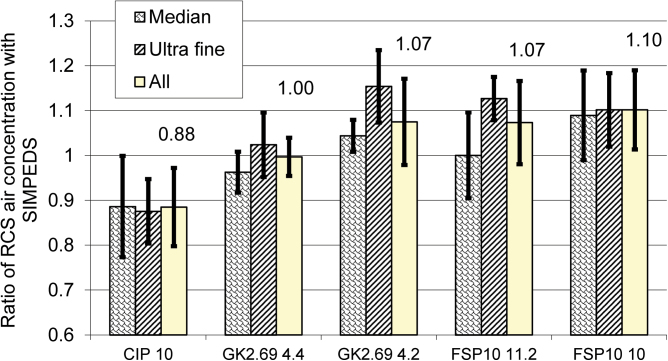
Ratio of RCS with SIMPEDS.

**Table 3.** Comparison of regression coefficients for respirable dust concentrations obtained for all samples and dusts

Sampler	Slope with SIMPEDS	Relative difference with Dorr Oliver (calculated)	Relative difference with Dorr Oliver (experimental, Lee *et al.*, 2012)
FSP10 (10 l·min^−1^)	*y* = **1.04** *x* (*r* ^2^ = 0.996)	1.26	
FSP10 (11.2 l·min^−1^)	*y* = **0.997** *x* (*r* ^2^ = 0.997)	1.21	1.19
GK2.69 (4.4 l·min^−1^)	*y* = **0.836** *x* (*r* ^2^ = 0.991)	1.03	1.06
GK2.69 (4.2 l·min^−1^)	*y* = **0.852** *x* (*r* ^2^ = 0.793)	1.01	
CIP 10 R	*y* = **0.836** *x* (*r* ^2^ = 0.950)	1.01	1.02
Dorr Oliver	*y* = **0.826** *x* (*r* ^2^ = 0.992)		

## RESULTS

The air concentration ranges for each sampler type collected for respirable dust were between 3.5 and 13 mg·m^−3^. Most air concentration values (>60%) were <5 mg·m^−3^. The range for GK 2.69 sampler at 4.2 l·min^−1^ is limited as it was not exposed to the very highest air concentrations, although it still covered the same range of mass loadings as the other samplers. Aerosols were sampled so that the filter loadings were between 180 and 1200 μg on the SIMPEDS sampler. The range of loadings, the repeatability of weighing the blank sampling medium, and the average standard deviation of the gravimetric weighing of the dust loading on three samples from each sampler type in each run are listed in Table 1.

**Table 1.** Loading ranges for respirable dust and standard deviation of weighing

Sampler	Collection medium	Pore size	Loading range (mg)	Repeatability standard deviation (μg) on blank	Average standard deviation (μg)
SIMPEDS	25-mm diameter silver filter	(0.8 μm)	0.18–1.0	1	11
FSP10	37-mm diameter cellulose nitrate filter	(8 μm)	1.3–5.2	15	27
GK2.69	37-mm diameter polyvinylchloride filter	(5 μm)	0.2–1.7	17	40
CIP 10 R	Polyurethane foam	n/a	1.0–4.3	143	148

n/a, not appropriate.

Included in the average standard deviation of weighing the deposited dust is the variability of the samplers and pumps. The variability of weighing three replicate loaded samples from each run for the CIP 10 R sampler is just as large as the repeatability when weighing the blank sampling medium itself. This indicates the major factor influencing the precision of this analysis is the variability of weighing the foam and cup sampling medium from the CIP 10 R.

### Differences between sampler collection efficiencies

The diagonal line in Table 2 lists the slope constants of the trend lines, calculated from the mixed effects models, comparing the mean air concentration of all samplers in each run with the measured air concentrations for each sampler of each sampler type and the 95% confidence interval. The slope constant is effectively the ratio of one sampler’s air concentration for respirable dust compared with the average value obtained by all samplers in each run over the range of respirable dust concentrations tested. The samplers are listed in order of relative difference for each ARD with the highest value on the left hand side of Table 2. The CIP 10 R sampler is the only sampler design that changes its position in ranking of slopes. The clear boxes contain the probability value (*P*) testing the inequality of the relative differences between two samplers with the Wald test. Only those probabilities where there was not a significant difference (*P* > 0.01) are shown since the probability values that indicate relationships between each sampler pair are less numerous.

**Table 2.** Slope values for respirable dust, 95% confidence intervals, and probability values comparing samplers

Sampler	Sampler (medium Arizona dust)					
	FSP10 10	FSP10 11.2	SIMPEDS	CIP 10 R	GK2.69 4.4	GK2.69 4.2
FSP10 10	1.12 (1.09–1.15)	*P* = 0.017				
FSP10 11.2		1.06 (1.03–1.09)	*P* = 1.000			
SIMPEDS			1.05 (1.02–1.07)			
CIP 10 R				0.95 (0.91–0.99)		*P* = 0.246
GK2.69 4.4					0.87 (0.85–0.88)	*P* = 1.000
GK2.69 4.2						0.87 (0.81–0.94)
**Sampler**	**Sampler (ultrafine Arizona dust)**					
	**FSP10 10**	**FSP10 11.2**	**SIMPEDS**	**GK2.69 4.2**	**GK2.69 4.4**	**CIP 10 R**
FSP10 10	1.11 (1.09–1.14)					
FSP10 11.2		1.04 (1.02–1.07)	*P* = 1.000			
SIMPEDS			1.04 (1.01–1.06)			
GK2.69 4.2				0.95 (0.93–0.98)	*P* = 0.122	*P* = 0.035
GK2.69 4.4					0.92 (0.90–0.94)	*P* = 0.720
CIP 10 R						0.86 (0.78–0.93)

Gravimetric differences between samplers when compared with the SIMPEDS when sampling the medium and ultrafine ARD are shown in Fig. 2. The slope values in Fig. 2 are not very different from the figures obtained by comparing the difference between the slope constants in the diagonal line shown in Table 2 for a sampler type and the SIMPEDS, so are not given additionally. For example, the difference in the slopes between SIMPEDS and the FSP10 at 10 l·min^−1^ for the ultrafine ARD is + 0.07 (1.11–1.04), so the slope shown in Fig. 2 for the FSP10 is 1.07. Fig. 2 shows the differences between the UK reference SIMPEDS sampler and each sampler type.

**2:**
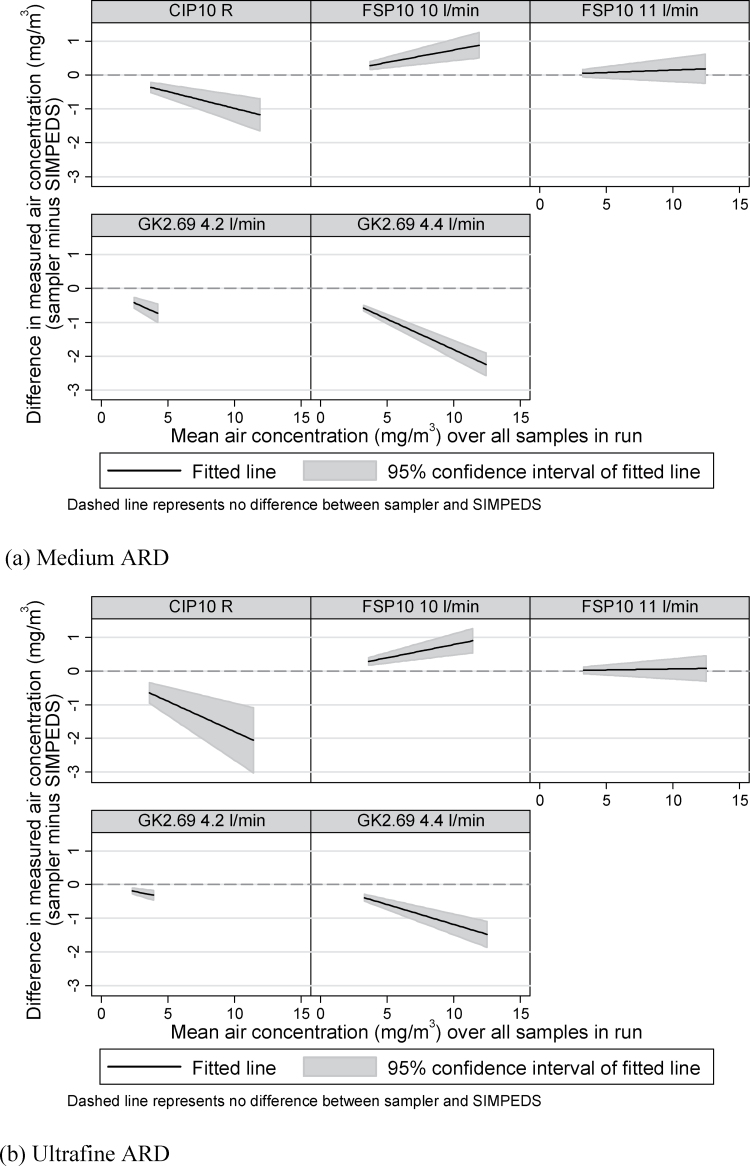
Difference in measured respirable dust concentration to the SIMPEDS for (a) medium ARD and (b) ultrafine ARD.

### Proportion of RCS within ARD

Fig. 3 compares the proportion of RCS measured by HSL sampled by each sampler design showing their selectivity for the quartz in each ARD. The CIP 10 R and the SIMPEDS measured the lowest proportions of quartz in these ARDs and the GK2.69 measured higher proportions. This made the air concentration data for RCS more comparable for the SIMPEDS and GK2.69 samplers as the values converged. The ratios for FSP10 at 10 l·min^−1^ and GK2.69 at 4.2 l·min^−1^ are slightly higher than those found in Stacey *et al.* (2013), which is possibly because they are not influenced by values obtained by other contributing laboratories in the earlier work. The ratios for the GK2.69 sampler at both flow rates are significantly different from the ratios obtained by the SIMPEDS (*t*-test *P* < 0.01).

The error bars on the chart in Fig. 3 represent 1 SD.

### Differences in RCS concentrations

Differences in RCS concentration between samplers and the SIMPEDS are shown in Fig. 4. Columns are shown for the ultrafine ARD, medium ARD, and all results for each sampler type and flow rate. The bars on the columns represent 1*σ* of ratios and provide an indication of the precision of RCS measurements relative to the SIMPEDS for each sampler type. The within run RCS results for the SIMPEDS sampler varied by an average of 3.7% (max = 7.6% and min = 0.5%). The variability of the ratios of the other samplers is a combination of the precision of the SIMPEDS and the sampler of interest. The results from these samplers are not significantly different from each other (*P* > 0.01) due to their variability. The variation of the ratios for each sampler type was <10% (range 4.2–9.8).

## DISCUSSION

Table 3 compares the differences in regression line slope coefficients, where the intercept is set to zero, for the air concentration of respirable dust collected by each sampler with the average value from the triplicate SIMPEDS samplers in each run over the range of air concentration values for both dust types and with those values obtained by Lee *et al.* (2012) when using the Dorr Oliver cyclone and two grades of coal dust. The mass median particle size of the aerosol of each coal dust in the work of Lee *et al.* (2012) was 2.33 and 4.48 μm and similar to those obtained for the aerosolized ARD in this work. The similarity of the mass median particle sizes of the ARD used in this work and the coal dust used by Lee *et al.* (2012) would lead to an expectation that these independent and separate comparisons of samplers would give equivalent results. Shown in Table 3 is the slope value for each sampler design calculated using the difference between the SIMPEDS and the Dorr Oliver cyclone (0.826) from data obtained in previous work (Stacey *et al.*, 2013). The slope for each sampler design was compared with the slope obtained for the Dorr Oliver and SIMPEDS to calculate the relative difference with the Dorr Oliver.

Slope values shown in Table 3 are slightly different from the values used in Fig. 2 as they are calculated in different ways. The slope coefficients presented here include all values from both types of dust. The regression values are extremely good (*r* ^2^ > 0.99) except with the CIP 10 R and GK2.69 at 4.2 l·min^−1^ samplers. The poorer regression value for the CIP 10 R is probably due to its apparent differing performance in the two ARD dust types shown in Table 2. The relative differences obtained from this work for the Dorr Oliver sampler are comparable with the work of Lee *et al.* (2012) indicating the aerosol generating systems at NIOSH and HSL obtain similar results when comparing the collection efficiencies of different sampler designs.

### Change in flow rate for GK2.69 and FSP10

In these tests there are three statistically distinct groups of performance for sampling respirable dust. These are Group 1: FSP10 at 10 l·min^−1^, Group 2: FSP10 at 11.2 l·min^−1^ and SIMPEDS, Group 3: GK2.69 (at 4.2 and 4.4 l·min^−1^) and the CIP 10 R.

### FSP10

The FSP10 was originally designed to collect dust to the British Medical Research Council definition for respirable dust with a mass median aerodynamic diameter of 5 μm at 9.2 l·min^−1^, which differs from the respirable dust specification described in ISO 7708 and EN 481, which has a mass median aerodynamic diameter of 4 μm. The FSP10 was also primarily designed for sampling inside vents rather than personal sampling (Cossey and Vaughan, 1987). Gravimetrically, the change in flow rate to 11.2 l·min^−1^ improves its comparability with the SIMPEDS for both ARD types (*P* = 1.000).

### GK 2.69

The small change in flow rate from 4.2 (recommended by the manufacturer) to 4.4 l·min^−1^ for the GK2.69 sampler makes very little difference in the mass collected in these tests. In addition, there seems to be a better relationship with the SIMPEDS when comparing RCS, rather than respirable dust concentrations, although this is also not significant. The lack of significant difference is not surprising since the change in recommended in flow rates is only 0.2 l·min^−1^, which is within the maximum deviation for flow rate change proposed in the guidance for RCS measurement in ISO 24095 (2009). That the GK2.69 seems to have a better comparability with the SIMPEDS when examining the results for RCS measurement from each sampler suggests it collects a larger proportion of crystalline silica in the respirable fraction of each ARD. This might be attributable to the apparently better fit to the ISO curve for respirable dust for the larger respirable-sized particles than other respirable samplers such as the FSP10 (Lee *et al.*, 2010).

### CIP 10 R

For the CIP 10 R, the results clearly show that although this sampler has a comparable performance with average air concentration for the median ARD (slope = 0.95), it does sample fewer small-sized respirable particles (-20%) when compared with the SIMPEDS. The undersampling of the smaller-sized respirable-sized particles is recorded in other work (Görner *et al.*, 2001). The CIP 10 R also appears to have a better fit to the ISO curve for the larger respirable particles (Lee *et al.*, 2010). The variability in weighing foams and plastic cups is a major factor affecting the variability of weighing respirable dust. However, it collects more dust than most other samplers described as respirable and the additional variability in absolute mass (μg) might not be significant in relative terms (%) when weighing milligrams. The CIP 10 R sampler did not seem to collect proportionally more RCS in the respirable dust in this study. Fig. 3 shows that the ratio of RCS in respirable dust for the CIP 10 R was comparable to that of the SIMPEDS sampler; however, it reported lower RCS values than the SIMPEDS (Fig. 4) because it collected a smaller mass of respirable dust (Table 2).

It is essential for an Occupational Hygienist to know that the sampler they use meets the ISO/CEN/ACIGH particle size selection criteria and it is also important to understand how it differs from other samplers, in terms of the mass collected, since this information has a direct practical benefit and aids the comparability of data. The aerosols used in this work are representative of a mineral dust containing a significant proportion of RCS, since it is the performance of samplers when measuring this hazardous chemical that interests us. Although correction factors might be proposed, and the results from this work seem consistent, it is not yet known if they are applicable in all circumstances, e.g. with different types of dusts. Adding a correction factor derived from our simple case may introduce an additional uncertainty. It is better that observed differences in performance are used to encourage Occupational Hygienists to select equipment or flow rates that have better comparability to a reference sampler, the ISO/CEN/ACIGH respirable convention or for manufacturers/researchers to ensure respirable samplers meet stringent performance criteria.

### CONCLUSIONS

The gravimetric differences obtained in this work compared favourably with values obtained at NIOSH. This indicates the separate systems used to evaluate the performance of samplers at these two national laboratories will give similar results when measuring the collection efficiencies of respirable samplers. The change in flow rates of the FSP10 to that recommended by NIOSH (11.2 l·min^−1^) produced a substantial improvement in the performance of the FSP10 sampler. The performance of the FSP10 was different from the SIMPEDS with a flow rate of 10 but not at 11.2 l·min^−1^, indicating a better comparability with the SIMPEDS sampler at the NIOSH-proposed flow rate. The change in flow rate to 11.2 l·min^−1^ for the FSP10 sampler should be a benefit in improving the precision of measurements at air concentrations lower than the present UK workplace exposure limit of 0.1 mg·m^−3^. The difference in flow rates for the GK2.69 sampler is small (0.2 l·min^−1^) and so was unlikely to make an impact in terms of a change in its collection efficiency. The performance of two different sampler types (GK2.69 versus FSP10) is different, although the average difference is less than ~14% with the NIOSH-proposed flow rates. These significant gravimetric differences indicate that it is useful for the Occupational Hygiene report to state the sampler type and flow rate used in the collection of samples, so an assessment of the potential difference with a reference sampler can be made to aid the comparability of data from different sources.

Variability from the small number of samples analysed (12 for each sampler) for RCS probably influenced the significance of any findings. The FSP10 produced more results comparable with the SIMPEDS for respirable dust than for RCS; however, the GK2.69 sampler at 4.4 l·min^−1^ obtained comparable RCS air concentration measurements for the types of ARD used in these tests, indicating it had sampled a larger proportion of crystalline silica in the respirable dust. The CIP 10 R tended to record lower values for both respirable dust and RCS. The CIP 10 R is known to undersample finer particulate with respect to ISO Standard recommendations for the penetration of particles to the alveolar region of the lungs, and has been suggested instead as a sampler that may better mimic deposition in that region.

## FUNDING

This publication and the work it describes were co-funded by the Health and Safety Executive in Great Britain (project JN 01110); the National Institute for Occupational Safety and Health in the USA.
